# Routine 36‐week scan: prediction of prolonged neonatal intensive care unit admission

**DOI:** 10.1002/uog.29267

**Published:** 2025-06-21

**Authors:** A. Arechvo, E. Demertzidou, S. Adjahou, A. Syngelaki, L. A. Magee, P. von Dadelszen, K. H. Nicolaides, R. Akolekar

**Affiliations:** ^1^ Fetal Medicine Research Institute, King's College Hospital London UK; ^2^ Institute of Women and Children's Health, School of Life Course and Population Sciences, King's College London London UK; ^3^ Fetal Medicine Unit, Medway Maritime Hospital, Gillingham UK; ^4^ Institute of Medical Sciences Canterbury Christ Church University Chatham UK

**Keywords:** adverse perinatal outcome, Doppler ultrasound, estimated fetal weight, fetal biometry, neonatal intensive care unit admission, pyramid of pregnancy care, third‐trimester screening

## Abstract

**Objectives:**

To examine the contribution of maternal demographic characteristics and elements of medical history to the prediction of prolonged (> 2 days) neonatal intensive care unit (NICU) admission for high‐dependency or intensive care, and to investigate the added value to such prediction of the findings from the 36‐week ultrasound scan and data from labor and delivery.

**Methods:**

We included 107 762 women with a singleton pregnancy who had undergone a routine ultrasound examination at 35 + 0 to 36 + 6 weeks' gestation. Assessment included medical history, estimated fetal weight (EFW), and Doppler measurements of uterine artery (UtA) pulsatility index (PI), umbilical artery (UA) PI and fetal middle cerebral artery (MCA) PI. Multivariable logistic regression was used to evaluate the independent contributions to prolonged NICU admission of maternal factors (demographic and pregnancy characteristics), findings from the 36‐week scan, and data from labor and delivery. Detection rates (DRs) and areas under the receiver‐operating‐characteristics curve (AUCs) for prolonged NICU admission were compared.

**Results:**

Overall, 946 (0.88%) neonates required prolonged NICU admission. The maternal factors that significantly contributed to prediction of prolonged NICU admission were body mass index, social deprivation, conception via *in‐vitro* fertilization, chronic hypertension, Type‐1 diabetes mellitus, pre‐eclampsia and gestational diabetes mellitus. Screening by maternal factors provided a DR of 20.7% at a false‐positive rate (FPR) of 10% (AUC, 0.606 (95% CI, 0.587–0.625)). Significant predictors from the 36‐week scan included deepest vertical pocket of amniotic fluid < 2 cm and ≥ 8 cm, UA‐PI >95^th^ percentile and MCA‐PI < 5^th^ percentile. Screening by a combination of maternal factors and 36‐week scan findings improved the DR at a 10% FPR to 27.5% (AUC, 0.637 (95% CI, 0.618–0.656)). When maternal factors, 36‐week scan findings, and data from labor and delivery (lower gestational age at delivery, delivery by Cesarean section and birth weight < 10^th^ and > 90^th^ percentile) were included in the model, the DR at a 10% FPR improved to 39.1% (AUC, 0.709 (95% CI, 0.690–0.728)). When this combined model was restricted to prediction of prolonged NICU admission for indications that excluded those affected by intrapartum events, the DR at a 10% FPR was 57.2% (AUC, 0.790 (95% CI, 0.764–0.815)).

**Conclusions:**

Maternal factors (particularly social deprivation), 36‐week scan findings, and data from labor and delivery each make an independent contribution to the risk of prolonged NICU admission at term. Nevertheless, more than 40% of these admissions are not predictable prior to labor and birth. © 2025 The Author(s). *Ultrasound in Obstetrics & Gynecology* published by John Wiley & Sons Ltd on behalf of International Society of Ultrasound in Obstetrics and Gynecology.

## INTRODUCTION

More than 600 000 neonates are born in England every year, of which approximately 10% are admitted to the neonatal care unit, for varying lengths of time, to receive special care (e.g. extra medical or nursing care), high‐dependency care (e.g. non‐invasive ventilation) or intensive care (24‐h care and close monitoring)^1^. More than 60% of neonates admitted to the neonatal care unit are born at term (≥ 37 weeks' gestation). Excluding fetal anomalies, the most commonly reported reasons for term admission are respiratory distress, infection, hypoglycemia, jaundice and asphyxia^2^, ^3^.

It is estimated that a proportion of term neonatal care unit admissions are predictable and avoidable. Prevention of admission would be of value to parents and neonatologists, to avoid separation of the mother from the neonate and thus disruption of bonding, and for establishment of breastfeeding^4^. It would also be of value to policymakers given the cost of neonatal care, even in the absence of neonatal morbidity with long‐term consequences^5^. These considerations are even more relevant for neonates requiring prolonged neonatal care, who are less likely to be admitted to the unit for only observation or septic workup. Nevertheless, there is no prediction model available for neonatal care unit admission or prolonged admission^6^.

There is good evidence that all pregnant women should be offered an ultrasound scan at 36 weeks' gestation, in addition to a routine ultrasound examination at 12 weeks' and 20 weeks' gestation. Such a late third‐trimester scan is useful for diagnosis of fetal abnormalities^7^, ^8^, prediction of small and large neonates^9^, ^10^, ^11^, diagnosis and management of non‐cephalic presentation^12^, assessment of the risk of pre‐eclampsia and the reduction of such risk by timed birth^13^, ^14^, ^15^, and prediction of adverse perinatal outcome^16^, ^17^.

The objectives of this study, which included 107 762 singleton pregnancies undergoing a routine ultrasound examination at 35 + 0 to 36 + 6 weeks' gestation, were to examine the contribution of maternal demographic characteristics and elements of medical history to the prediction of prolonged (> 2 days) neonatal intensive care unit (NICU) admission for high‐dependency or intensive care, and to investigate the added value to such prediction of the findings from the 36‐week scan and data from labor and delivery.

## METHODS

### Study population and design

This was a retrospective analysis of data collected as part of a prospective cohort study of women with a singleton pregnancy who had undergone a routine ultrasound examination at 35 + 0 to 36 + 6 weeks' gestation (i.e. a 36‐week scan) at King's College Hospital, London or Medway Maritime Hospital, Gillingham, UK, between March 2014 and November 2023. In the participating hospitals, all women with a singleton pregnancy are offered a routine obstetric ultrasound examination at 11 + 0 to 13 + 6 weeks' and 19 + 0 to 23 + 6 weeks' gestation.

During the visit at 35 + 0 to 36 + 6 weeks, we recorded maternal demographics and medical history, and carried out an ultrasound examination which included: determination of fetal presentation and placental position; detailed assessment of the fetal anatomy^8^; measurement of fetal head circumference, abdominal circumference and femur length^18^ to calculate estimated fetal weight (EFW) using the formula of Hadlock *et al*.^19^, which was identified in a systematic review as the most accurate model for EFW^20^; measurement of the deepest vertical pocket of amniotic fluid; and Doppler assessment of uterine artery (UtA) pulsatility index (PI), umbilical artery (UA) PI and fetal middle cerebral artery (MCA) PI, as well as calculation of the cerebroplacental ratio (CPR) as the ratio of MCA‐PI to UA‐PI^21^, ^22^. Gestational age was determined by measurement of the fetal crown–rump length at 11 + 0 to 13 + 6 weeks^23^. Ultrasound examinations were carried out by examiners who had obtained the Fetal Medicine Foundation (FMF) certificate of competence in ultrasound examination for fetal abnormalities.

The inclusion criteria for this study were singleton pregnancy delivering a liveborn neonate ≥ 35 + 0 weeks' gestation. We excluded pregnancies with aneuploidy, those ending in stillbirth and those with major fetal abnormality, defined as a defect requiring postnatal surgery and/or associated with severe physical or intellectual disability. The study entitled “Prediction of pregnancy complications” was approved by the NHS Research Ethics Committee (No.: 02‐03‐033), and all women provided written informed consent for participation. The outcome was evaluated in a blinded manner by clinicians who were not involved in managing the patients (as expected in the Newcastle–Ottawa scale for risk of bias).

### Outcome measures

The primary outcome measure of interest was prolonged admission (> 2 days) to the neonatal care unit for high‐dependency or intensive care (such as total parenteral nutrition or invasive ventilation, respectively), termed prolonged NICU admission. We excluded admissions for special care, as these neonates require additional medical or nursing care but are usually hemodynamically stable. Prolonged NICU admissions were stratified by the primary indication for admission, and a nested cohort of prolonged NICU admissions that excluded those affected by intrapartum events and those with indications that could not be detected by maternal factors, findings from the 36‐week scan and data from labor and delivery, was selected as the outcome measure.

Data on baseline maternal demographics (including self‐declared ethnicity), pregnancy characteristics (including hypertensive disorders of pregnancy and gestational diabetes mellitus (GDM)) and pregnancy outcomes were collected from the hospital maternity records. Other outcomes included gestational age at delivery, method of onset of labor and delivery, and birth weight. The index of multiple deprivation (IMD) was used to assess weighted relative deprivation based on seven domains: income, employment, education, health, crime, housing and living environment^24^. The IMD score was divided into five equal quintiles, with the 1^st^ quintile being the most deprived and the 5^th^ being the least deprived. EFW and birth‐weight percentiles were derived from the FMF fetal and neonatal population weight charts, and a small‐for‐gestational‐age (SGA) fetus or neonate was defined as EFW or birth weight < 10^th^ percentile, respectively, while a large‐for‐gestational‐age (LGA) fetus or neonate was defined as EFW or birth weight > 90^th^ percentile, respectively^25^. Pre‐eclampsia was defined according to the American College of Obstetricians and Gynecologists^26^.

### Statistical analysis

Data were expressed as median (interquartile range (IQR)) and *n* (%) for categorical variables. Comparisons of groups to assess significant differences were performed using the Mann–Whitney *U*–test for continuous variables, and the chi‐square test or Fisher's exact test for categorical variables. Significance was assumed at 5%.

The following steps were undertaken for the statistical analysis. First, continuous variables were centered by subtracting the arithmetic mean from each value to avoid effects of multicollinearity in regression analyses. Categorical variables were dummy coded as binary or ordinal variables, to estimate the independent effect of each category. Univariable and multivariable logistic regression analysis, with backward stepwise elimination including only variables significant in the univariable analysis, were undertaken to determine which of the factors (from maternal demographics, including IMD, obstetric and medical history, and pregnancy complications) provided a significant contribution to the prediction of prolonged NICU admission (Model 1). Prior to multivariable analysis, the relationship between dependent and continuous variables was examined to assess whether the association was linear or non‐linear.

Second, predicted risks from the multivariable regression analysis for maternal and pregnancy factors known before the 36‐week scan (i.e. maternal demographics, obstetric and medical history, and pregnancy characteristics) were logarithmically transformed to derive the maternal factor *a‐priori* risk (logit); this was calculated using the formula: risk = odds/(1 + odds), where odds = e^Y^ and Y was the linear predictor derived from multivariate logistic regression analysis. Univariable and multivariable regression analyses were undertaken by entering the maternal factor‐derived *a‐priori* risk (logit) and findings from the 36‐week scan, to assess which findings from the 36‐week scan provided a significant contribution to the prediction of prolonged NICU admission (Model 2).

Third, data from labor and delivery were entered into the univariable and multivariable regression analysis to examine which factors were significant independent predictors of prolonged NICU admission (Model 3), and whether they improved the performance of screening provided by maternal factors (logit, as above) and the 36‐week scan findings.

Finally, the distribution of risks for each of the multivariable regression models was used to calculate detection rates (DRs) at fixed false‐positive rates (FPRs), based on area under the receiver‐operating‐characteristics curve (AUC) analysis. The screening performance of different models was assessed by comparison of AUCs, using *Z*‐statistics.^27^

In sensitivity analyses, each of the three models was applied to a nested cohort of cases of prolonged NICU admission for indications that are potentially predictable from the available maternal factors, findings from the 36‐week scan and data from labor and delivery. We excluded those affected by intrapartum events such as poor condition at birth (as this often reflects management during labor and delivery), sepsis (as this is secondary to intrapartum maternal pyrexia, which may or may not be secondary to preterm prelabor rupture of membranes) and jaundice or hemolytic disease (as this depends on blood group and neonatal physiological factors, as well as breastfeeding).

The statistical software packages SPSS version 29.0 (IBM SPSS Statistics for Windows; IBM Corp., Armonk, NY, USA) and Medcalc (Medcalc Software, Mariakerke, Belgium) were used for data analysis.

## RESULTS

### Patient characteristics

The study population of 107 762 singleton pregnancies included 946 (0.88%) which delivered a neonate requiring prolonged NICU admission and 106 816 which did not. There were five primary indications for prolonged NICU admission: poor condition at birth requiring invasive cardiorespiratory support (412/946; 43.6%), respiratory distress syndrome (386/946; 40.8%), hypoglycemia (65/946; 6.9%), suspected sepsis (62/946; 6.6%) and hemolytic disease/jaundice (21/946; 2.2%).

Characteristics of the study population are shown in Table 1. There were significant differences between the two groups in many maternal and pregnancy characteristics, 36‐week scan findings and pregnancy outcomes. In pregnancies that delivered a neonate requiring prolonged NICU admission compared with those that did not: median maternal body mass index (BMI) was higher; women were more often of Black or ‘other’ non‐White ethnicity; women were more often in the 1^st^ IMD quintile (most deprived); a greater number of pregnancies were conceived via *in‐vitro* fertilization (IVF); maternal comorbidities (chronic hypertension and Type‐1 or Type‐2 pre‐existing diabetes mellitus) were more prevalent; and pregnancy was more often complicated by gestational hypertension, pre‐eclampsia or GDM. Additionally, in pregnancies that delivered a neonate requiring prolonged NICU admission compared with those that did not: more pregnancies had EFW < 10^th^ percentile and > 90^th^ percentile; the deepest vertical pocket of amniotic fluid was more often reduced or increased; UtA‐PI and UA‐PI were more often elevated; and MCA‐PI and CPR were more often reduced. In terms of pregnancy outcomes, gestational age at delivery was lower (difference in medians of 0.9 weeks), vaginal delivery was less common, elective and emergency Cesarean section were more common and newborns were more frequently SGA or LGA, in pregnancies that delivered a neonate requiring prolonged NICU admission compared with those that did not.

**Table 1:** Maternal and pregnancy characteristics in pregnancies with and those without prolonged neonatal intensive care unit (NICU) admission*

Characteristic	No NICU admission (*n* = 106 816)	NICU admission (*n* = 946)	*P*
*Maternal characteristics*			
Age (years)	32.0 (27.9–35.6)	31.9 (27.5–36.0)	0.690
Body mass index (kg/m^2^)	29.1 (26.1–33.0)	30.5 (27.1–34.9)	< 0.001
Smoker	6790 (6.4)	71 (7.5)	0.150
Ethnicity			
White	80 058 (74.9)	710 (75.1)	0.938
Black	15 472 (14.5)	160 (16.9)	0.035
South Asian	5906 (5.5)	44 (4.7)	0.239
Other	5380 (5.1)	32 (3.4)	0.025
Index of multiple deprivation			
Quintile 5 (least deprived)	13 238 (12.4)	73 (7.7)	< 0.001
Quintile 4	17 817 (16.7)	149 (15.8)	0.524
Quintile 3	25 280 (23.7)	214 (22.6)	0.542
Quintile 2	29 796 (27.9)	277 (29.3)	0.306
Quintile 1 (most deprived)	20 655 (19.3)	233 (24.6)	< 0.001
Conceived via *in‐vitro* fertilization	3956 (3.7)	55 (5.8)	< 0.001
Obstetric history			
Nulliparous	49 028 (45.9)	456 (48.2)	0.158
Parous, no previous SGA or PE	47 614 (44.6)	393 (41.5)	0.062
Parous, previous SGA or PE	10 174 (9.5)	97 (10.3)	0.447
Pre‐existing medical condition			
Chronic hypertension	1233 (1.2)	25 (2.6)	< 0.001
DM Type 1	417 (0.4)	20 (2.1)	< 0.001
DM Type 2	795 (0.7)	14 (1.5)	0.009
Obstetric complication			
Gestational hypertension	2689 (2.5)	34 (3.6)	0.036
PE	2808 (2.6)	56 (5.9)	< 0.001
Gestational DM	7144 (6.7)	93 (9.8)	< 0.001
*Findings from 36‐week scan*			
EFW			
10–90^th^ percentile	88 165 (82.5)	652 (68.9)	< 0.001
< 10^th^ percentile	8410 (7.9)	137 (14.5)	< 0.001
> 90^th^ percentile	10 241 (9.6)	157 (16.6)	< 0.001
Amniotic fluid DVP			
2–7 cm	103 657 (97.0)	868 (91.8)	< 0.001
< 2 cm	258 (0.2)	17 (1.8)	< 0.001
≥ 8 cm	2901 (2.7)	61 (6.4)	< 0.001
UtA‐PI > 95^th^ percentile	8110 (7.6)	108 (11.4)	< 0.001
UA‐PI > 95^th^ percentile	2604 (2.4)	67 (7.1)	< 0.001
MCA‐PI < 5^th^ percentile	2872 (2.7)	61 (6.4)	< 0.001
CPR < 5^th^ percentile	4461 (4.2)	91 (9.6)	< 0.001
*Pregnancy outcomes*			
Gestational age at delivery (weeks)	39.9 (39.0–40.7)	39.0 (37.3–40.4)	< 0.001
Mode of delivery			
Vaginal	73 283 (68.6)	417 (44.1)	< 0.001
Emergency CS (fetal distress)	6738 (6.3)	153 (16.2)	< 0.001
Emergency CS (other indications)	10 752 (10.1)	115 (12.2)	0.032
Elective CS (SGA/FGR)	604 (0.6)	37 (3.9)	< 0.001
Elective CS (other indications)	15 439 (14.5)	224 (23.7)	< 0.001
Birth weight			
10–90^th^ percentile	84 653 (79.3)	604 (63.8)	< 0.001
< 10^th^ percentile	12 270 (11.5)	185 (19.6)	< 0.001
> 90^th^ percentile	9893 (9.3)	157 (16.6)	< 0.001

Data are given as median (interquartile range) or *n* (%). Data on index of multiple deprivation were not available in all cases.

* Prolonged NICU admission defined as admission for > 2 days for high‐dependency or intensive care, not including special care. CPR, cerebroplacental ratio; CS, Cesarean section; DM, diabetes mellitus; DVP, deepest vertical pocket; EFW, estimated fetal weight; FGR, fetal growth restriction; MCA, middle cerebral artery; PE, pre‐eclampsia; PI, pulsatility index; SGA, small‐for‐gestational age; UA, umbilical artery; UtA, uterine artery.

**Figure 1:**
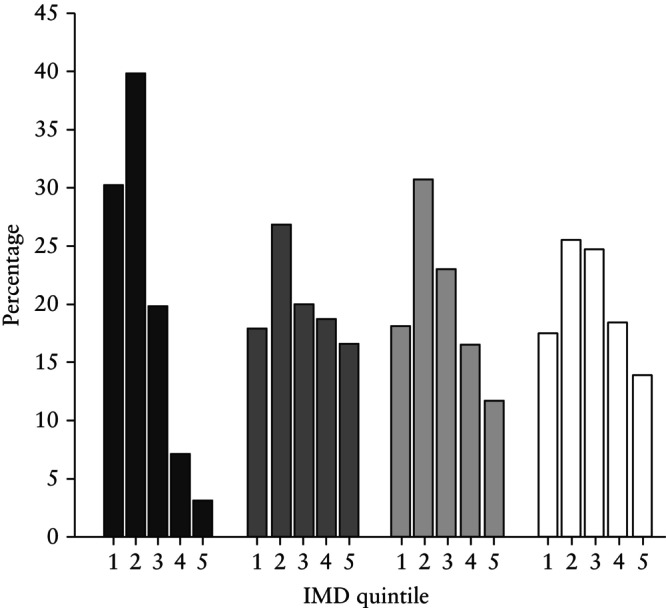
Distribution of index of multiple deprivation (IMD) quintiles stratified by maternal ethnicity. 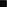, Black ethnicity; 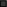, South Asian ethnicity; 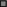, other ethnicity; 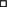, White ethnicity.

The distribution of IMD quintiles stratified by ethnicity is shown in Figure 1. Compared with White women (43.0% (34 725/80 768)), there were significantly more women in the 1^st^ and 2^nd^ IMD quintiles that were Black (70.0% (10 936/15 632)), South Asian (44.7% (2659/5950)) or of ‘other’ ethnicity (48.8% (2641/5412)) (*P* < 0.001 for all).

### Model 1: Screening by maternal and pregnancy characteristics

**Figure 2:**
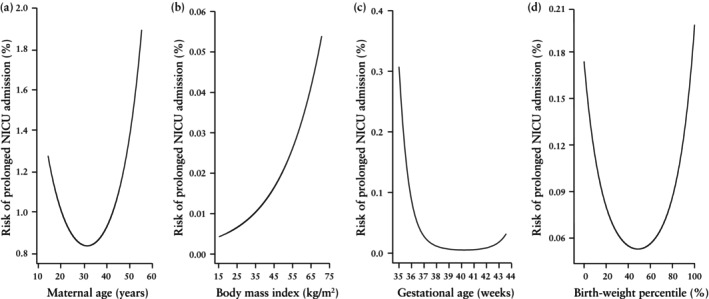
Association between risk of prolonged neonatal intensive care unit (NICU) admission and (a) maternal age, (b) maternal body mass index, (c) gestational age at delivery and (d) birth‐weight percentile.

Univariable logistic regression analysis demonstrated that there was a significant contribution to the prediction of prolonged NICU admission from maternal age, BMI, ethnicity, IMD, conception via IVF, pre‐existing medical complications (chronic hypertension and diabetes mellitus Type 1 and 2), and obstetric complications (gestational hypertension, pre‐eclampsia and GDM) (Table 2). Maternal age had a non‐linear U‐shaped association with risk of prolonged NICU admission, whereas BMI had a monotone relationship (Figures 2a and 2b, respectively).

**Table 2:** Univariable and multivariable logistic regression analysis for prediction of prolonged neonatal intensive care unit (NICU) admission* from maternal and pregnancy characteristics

	Univariable		Multivariable	
Characteristic	OR (95% CI)	*P*	aOR (95% CI)	*P*
Intercept	—	—	−5.310	—
Maternal age	0.91 (0.83–0.99)	0.046	—	—
Maternal age^2^	1.01 (1.00–1.03)	0.047	—	—
Body mass index – 30 (in kg/m^2^)	1.05 (1.04–1.06)	< 0.001	1.04 (1.03–1.05)	< 0.001
Smoker	1.20 (0.94–1.52)	0.150	—	—
Ethnicity		0.018		
White	Reference		—	
Black	1.17 (0.98–1.39)	0.081	—	—
South Asian	0.84 (0.62–1.14)	0.264	—	—
Other	0.67 (0.47–0.96)	0.028	—	—
Index of multiple deprivation		< 0.001		< 0.001
Quintile 5 (least deprived)	Reference		Reference	
Quintile 4	1.52 (1.15–2.01)	0.003	1.53 (1.16–2.03)	0.003
Quintile 3	1.54 (1.18–2.01)	0.002	1.55 (1.18–2.02)	0.001
Quintile 2	1.69 (1.31–2.19)	< 0.001	1.65 (1.27–2.14)	< 0.001
Quintile 1(most deprived)	2.05 (1.58–2.67)	< 0.001	1.96 (1.50–2.56)	< 0.001
Conceived via *in‐vitro* fertilization	1.61 (1.22–2.11)	< 0.001	1.71 (1.30–2.25)	< 0.001
Obstetric history		0.171		
Nulliparous	Reference			
Parous, no previous PE or SGA	0.89 (0.78–1.02)	0.084	—	—
Parous, previous PE or SGA	1.03 (0.82–1.28)	0.825	—	—
Pre‐existing medical condition				
Chronic hypertension	2.32 (1.56–3.47)	< 0.001	1.52 (1.01–2.29)	0.048
DM Type 1	5.55 (3.53–8.74)	< 0.001	5.13 (3.25–8.10)	< 0.001
DM Type 2	2.04 (1.20–3.47)	0.009	—	—
Obstetric complication				
Gestational hypertension	1.44 (1.02–2.04)	0.037	—	—
PE	2.33 (1.77–3.06)	< 0.001	1.84 (1.39–2.43)	< 0.001
Gestational DM	1.52 (1.23–1.89)	< 0.001	1.30 (1.04–1.62)	0.019

* Prolonged NICU admission defined as admission for > 2 days for high‐dependency or intensive care, not including special care. aOR, adjusted odds ratio; DM, diabetes mellitus; OR, odds ratio; PE, pre‐eclampsia; SGA, small‐for‐gestational age.

**Figure 3:**
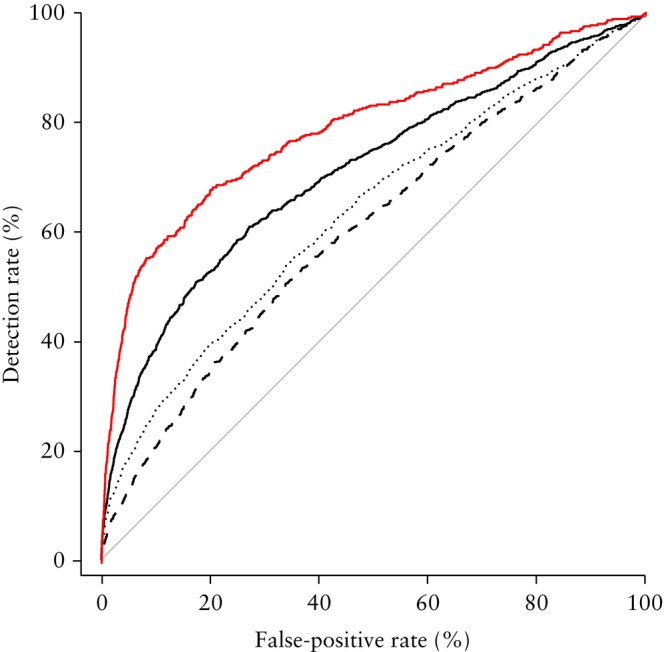
Receiver‐operating‐characteristics curves for predicition of prolonged neonatal intensive care unit (NICU) admission, based on: maternal and pregnancy characteristics (maternal factors; Model 1) (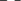); maternal factors and findings from the 36‐week scan (Model 2) (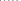); maternal factors, findings from the 36‐week scan, and data from labor and delivery (combined test; Model 3) (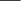); and the combined test in a nested cohort of prolonged NICU admissions for respiratory distress syndrome or hypoglycemia (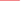).

Multivariable logistic regression analysis demonstrated that factors providing a significant contribution to the prediction of prolonged NICU admission were maternal BMI, IMD, conception via IVF, chronic hypertension, Type‐1 diabetes mellitus, pre‐eclampsia and GDM (*R* ^2^ = 0.017; *P* < 0.001) (Table 2). Screening by maternal factors and pregnancy characteristics provided a DR of 20.7% for a FPR of 10% (AUC, 0.606 (95% CI, 0.587–0.625)) (Figure 3).

### Model 2: Screening by maternal factors and findings from the 36‐week scan

The maternal factor‐derived *a‐priori* risk was calculated from the abovementioned factors found to be significant in multivariable regression analysis. The formula used for the calculation of the linear predictor was: Y = −5.310 + (0.039 × BMI) + (0.672 × IMD quintile 1) + (0.499 × IMD quintile 2) + (0.435 × IMD quintile 3) + (0.425 × IMD quintile 4) + (0.536 × IVF conception) + (0.417 × chronic hypertension) + (1.634 × diabetes mellitus Type 1) + (0.608 × pre‐eclampsia) + (0.262 × GDM).

Univariable and multivariable logistic regression analyses demonstrated that, in the prediction of prolonged NICU admission, there was a significant contribution from maternal factor‐derived *a‐priori* risk (logit), EFW (< 10^th^ percentile and > 90^th^ percentile), amniotic fluid volume (reduced and increased), UA‐PI > 95^th^ percentile, MCA‐PI < 5^th^ percentile and CPR < 5^th^ percentile (*R* ^2^ = 0.035; *P* < 0.001) (Table 3). Screening by maternal factors and findings from the 36‐week scan provided a DR of 27.5% for a FPR of 10% (AUC, 0.637 (95% CI, 0.618–0.656)), which was significantly higher than that provided by maternal factors alone (*Z‐*statistic, −2.192; *P* = 0.028) (Figure 3).

**Table 3:** Univariable and multivariable logistic regression analysis for prediction of prolonged neonatal intensive care unit (NICU) admission* from log_10_ transformed maternal and pregnancy characteristics (logit) and findings from 36‐week scan

	Univariable analysis		Multivariable analysis	
Characteristic	OR (95% CI)	*P*	aOR (95% CI)	*P*
Logit (log_10_ *a‐priori* risk)	10.41 (7.58–14.29)	< 0.001	8.02 (5.80–11.10)	< 0.001
*Findings from 36‐week scan*				
EFW		< 0.001		< 0.001
10^th^–90^th^ percentile	Reference		Reference	
< 10^th^ percentile	2.20 (1.83–2.65)	< 0.001	1.85 (1.52–2.26)	< 0.001
> 90^th^ percentile	2.07 (1.74–2.47)	< 0.001	1.58 (1.31–1.90)	< 0.001
Amniotic fluid DVP		< 0.001		< 0.001
2–7 cm	Reference		Reference	
< 2 cm	7.87 (4.80–12.91)	< 0.001	4.35 (2.57–7.35)	< 0.001
≥ 8 cm	2.51 (1.93–3.26)	< 0.001	1.86 (1.41–2.44)	< 0.001
UtA‐PI > 95^th^ percentile	1.57 (1.28–1.92)	< 0.001	—	—
UA‐PI > 95^th^ percentile	3.05 (2.37–3.92)	< 0.001	1.83 (1.35–2.48)	< 0.001
MCA‐PI < 5^th^ percentile	2.49 (1.92–3.24)	< 0.001	1.66 (1.23–2.24)	< 0.001
CPR < 5^th^ percentile	2.44 (1.96–3.04)	< 0.001	1.38 (1.04–1.84)	0.025

* Prolonged NICU admission defined as admission for > 2 days for high‐dependency or intensive care, not including special care. aOR, adjusted odds ratio; CPR, cerebroplacental ratio; EFW, estimated fetal weight; MCA, middle cerebral artery; OR, odds ratio; PI, pulsatility index; UA, umbilical artery; UtA, uterine artery.

### Model 3: Screening by maternal factors, findings from the 36‐week scan and data from labor and delivery

Univariable logistic regression analysis demonstrated that, in the prediction of prolonged NICU admission, there was a significant contribution from maternal factor‐derived *a‐priori* risk (logit), EFW (< 10^th^ percentile and > 90^th^ percentile), amniotic fluid volume (reduced and increased), fetoplacental Doppler parameters (UtA‐PI and UA‐PI > 95^th^ percentile, and MCA‐PI and CPR < 5^th^ percentile), lower gestational age at delivery (Figure 2c), mode of delivery (elective and emergency Cesarean section) and birth‐weight percentile (SGA and LGA) (Table 4). There was a non‐linear U‐shaped association between birth‐weight percentile and risk of prolonged NICU admission (Figure 2d).

**Table 4:** Univariable and multivariable logistic regression analysis for prediction of prolonged neonatal intensive care unit (NICU) admission* from log_10_ transformed maternal and pregnancy characteristics (logit), findings from 36‐week scan and data from labor and delivery

	Univariable analysis		Multivariable analysis	
Combined model	OR (95% CI)	*P*	aOR (95% CI)	*P*
Logit (log_10_ *a‐priori* risk)	10.41 (7.58–14.29)	< 0.001	3.09 (2.22–4.30)	< 0.001
*Findings from 36‐week scan*				
EFW		< 0.001		
10^th^–90^th^ percentile	Reference			
< 10^th^ percentile	2.20 (1.83–2.65)	< 0.001	—	—
> 90^th^ percentile	2.07 (1.74–2.47)	< 0.001	—	—
Amniotic fluid DVP		< 0.001		< 0.001
2–7 cm	Reference		Reference	
< 2 cm	7.87 (4.80–12.91)	< 0.001	2.23 (1.31–3.79)	0.003
≥ 8 cm	2.51 (1.93–3.26)	< 0.001	1.51 (1.15–1.98)	0.003
UtA‐PI > 95^th^ percentile	1.57 (1.28–1.92)	< 0.001	—	—
UA‐PI > 95^th^ percentile	3.05 (2.37–3.92)	< 0.001	1.42 (1.07–1.88)	0.016
MCA‐PI < 5^th^ percentile	2.49 (1.92–3.24)	< 0.001	1.51 (1.14–2.00)	0.004
CPR < 5^th^ percentile	2.44 (1.96–3.04)	< 0.001	—	—
*Data from labor and delivery*				
Gestational age at delivery	0.63 (0.60–0.65)	< 0.001	0.70 (0.67–0.74)	< 0.001
Mode of delivery		< 0.001		< 0.001
Vaginal	Reference		Reference	
Emergency CS (fetal distress)	3.99 (3.31–4.81)	< 0.001	3.63 (3.00–4.40)	< 0.001
Emergency CS (other indications)	1.88 (1.53–2.31)	< 0.001	1.55 (1.25–1.91)	< 0.001
Elective CS (SGA)	10.77 (7.62–15.21)	< 0.001	2.88 (1.93–4.31)	< 0.001
Elective CS (other indications)	2.55 (2.17–3.00)	< 0.001	1.72 (1.45–2.04)	< 0.001
Birth weight		< 0.001		< 0.001
10–90^th^ percentile	Reference		Reference	
< 10^th^ percentile	2.11 (1.79–2.49)	< 0.001	1.25 (1.04–1.52)	0.021
> 90^th^ percentile	2.22 (1.86–2.65)	< 0.001	1.75 (1.45–2.10)	< 0.001

* Prolonged NICU admission defined as admission for > 2 days for high‐dependency or intensive care, not including special care. aOR, adjusted odds ratio; CPR, cerebroplacental ratio; CS, Cesarean section; DVP, deepest vertical pocket; EFW, estimated fetal weight; MCA, middle cerebral artery; OR, odds ratio; PI, pulsatility index; SGA, small‐for‐gestational age; UA, umbilical artery; UtA, uterine artery.

Multivariable logistic regression analysis demonstrated that there was significant prediction of prolonged NICU admission from all factors which were significant in the univariable analysis, with the exception of EFW (*P =* 0.885), UtA‐PI > 95^th^ percentile (*P =* 0.808) and CPR < 5^th^ percentile (*P =* 0.342) (*R* ^2^ = 0.073; *P* < 0.001) (Table 4). This model provided a DR of 39.1% for a FPR of 10% (AUC, 0.709 (95% CI, 0.690–0.728)), which was significantly higher than that provided by either maternal factors alone (*Z*‐statistic, −7.283; *P* < 0.001) or the combination of maternal factors and findings from the 36‐week scan (*Z‐*statistic, −5.091; *P* < 0.001) (Figure 3).

### Sensitivity analysis

The nested cohort included those with prolonged NICU admission for respiratory distress syndrome or hypoglycemia (*n* = 451). In the sensitivity analysis in the selected cohort, the DR at a 10% FPR in Model 1 was 22.4% (AUC, 0.616 (95% CI, 0.589–0.643)), in Model 2 it was 34.4% (AUC, 0.676 (95% CI, 0.649–0.703)) and in Model 3 it was 57.2% (AUC, 0.790 (95% CI, 0.764–0.815)). Therefore, there was a negligible improvement in DR when screening was based on maternal factors alone (20.7% to 22.4%), a small improvement in DR when including the findings from the 36‐week scan (27.5% to 34.4%) and a substantial improvement in DR with inclusion of both findings from the 36‐week scan and data from labor and delivery (39.1% to 57.2%).

## DISCUSSION

### Main findings

There are four main findings of this study of 107 762 women of a socioeconomically and ethnically diverse population with a singleton pregnancy attending for a routine 36‐week scan. First, there were a greater number of neonates that required prolonged NICU admission born to women living in the most (*vs* least) deprived areas and Black women. However, in multivariable analysis for prediction of prolonged NICU admission, there was an independent contribution only from IMD, and not from ethnicity.

Second, prediction of prolonged NICU admission that was achieved by maternal characteristics and medical and obstetric history was significantly improved by addition of findings from the 36‐week scan. Significant contributors were EFW < 10^th^ percentile and > 90^th^ percentile, deepest vertical pocket of amniotic fluid < 2 cm and ≥ 8 cm, and Doppler findings of UA‐PI > 95^th^ percentile, MCA‐PI < 5^th^ percentile and CPR < 5^th^ percentile.

Third, further improvement on the prediction of prolonged NICU admission was achieved by including data from labor and delivery. Significant contributors were low gestational age at delivery, emergency Cesarean section for fetal distress or other indications, elective Cesarean section for SGA or other indications, and birth weight < 10^th^ or > 90^th^ percentile.

Fourth, prediction of prolonged NICU admission by a combination of maternal factors, findings from the 36‐week scan, and data from labor and delivery was poor. The estimated DR was 39% for a FPR of 10%. However, the DR improved to 57% after excluding cases with indications for prolonged NICU admission that could not be predicted by maternal factors, findings from the 36‐week scan and data from labor and delivery, and those that could be affected by intrapartum events.

### Comparison with results of previous studies

We evaluated prolonged NICU admission, given that it has the greatest implications for parents and healthcare costs. As such, it is unsurprising that the incidence of prolonged NICU admission was low, at just under 1%, compared with a prior estimate of 7% in the UK which included all NICU admissions (i.e. for special care as well as high‐dependency and intensive care) of any duration^6^.

The result that IMD, but not ethnicity, was independently associated with prolonged NICU admission is similar to our findings for the prediction of pre‐eclampsia^28^. This suggests that the relationship between Black ethnicity and prolonged NICU admission may be mediated by greater socioeconomic deprivation and other associated factors in this group, such as higher BMI, higher prevalence of chronic hypertension and higher incidence of pre‐eclampsia.

There is no predictive model available for the risk of prolonged NICU admission for term singletons. Similar to our study, prior research has found significant associations between neonatal care unit admission, or NICU admission specifically, and near‐term gestational age at delivery, low and high birth weight, and decreased CPR^29^, ^30^, ^31^, in addition to characteristics of the hospital in which delivery occurred which were more important than individual characteristics^32^. As such, it is unsurprising that in our study, the 36‐week scan findings added value to the prediction of prolonged NICU admission, expanding the usefulness of this 36‐week assessment to birth planning^7^, ^8^, ^9^, ^10^, ^11^, ^12^, ^13^, ^14^, ^15^, ^16^. While the magnitude of prediction was modest, it should be noted that clinicians were not masked to the 36‐week scan findings and were at liberty to use these to inform care, such as elective Cesarean delivery for EFW < 3^rd^ percentile. Consequently, the importance of the 36‐week scan is likely to have been underestimated in our study.

Nevertheless, before membrane rupture or onset of labor, our findings illustrate that most prolonged NICU admissions are not predictable based on routinely available variables and, even when analyses were restricted to those admissions theoretically predictable by maternal factors or 36‐week scan findings, more than 40% of prolonged NICU admissions were not predictable. This is not surprising, given biological variability, the complex interplay of maternal and fetoplacental factors and the dynamic nature of labor. These data should be included in informed decision‐making with regards to place of birth, given the importance of continuous monitoring and ready access to skilled maternity‐care providers.

### Strengths and limitations

The strengths of this study include, first, prospective examination of a large population of women with a singleton pregnancy attending for routine pregnancy care at 35–37 weeks' gestation and recording of maternal and pregnancy characteristics that are known to be associated with development of pregnancy complications; second, examination of the independent effects of maternal ethnicity and social deprivation on prolonged NICU admission; and third, examination of the additive value (to maternal factors alone) of both findings from the 36‐week scan and data from labor and delivery, on the prediction of prolonged NICU admission.

There are some limitations of this study. First, whilst IMD is the standard measure of social deprivation in the UK, it is not an individual‐level measure as it is based on the postcode of residence; not everyone living in a highly deprived area is necessarily deprived, and some individuals who are deprived may reside in areas with lower levels of deprivation. Second, data that may be important for prediction of prolonged NICU stay were not available, including maternal mental health problems (which may impact on breastfeeding), medications (antepartum and intrapartum, including analgesia/anesthesia), information about infection in labor (by oral temperature) and operative vaginal delivery.

### Conclusions

Maternal factors (particularly social deprivation), 36‐week scan findings, and data from labor and delivery each make an independent contribution to the risk of prolonged NICU admission at term. While the absolute rate is low, most such admissions are not predictable based on commonly available factors. These findings should inform shared decision‐making regarding the place of birth.

## Data Availability

Research data are not shared.
